# Camera Sensor Arrangement for Crop/Weed Detection Accuracy in Agronomic Images

**DOI:** 10.3390/s130404348

**Published:** 2013-04-02

**Authors:** Juan Romeo, José Miguel Guerrero, Martín Montalvo, Luis Emmi, María Guijarro, Pablo Gonzalez-de-Santos, Gonzalo Pajares

**Affiliations:** 1 Department of Software Engineering and Artificial Intelligence, Faculty of Informatics, Complutense University, Madrid 28040, Spain; E-Mails: jmguerre@fdi.ucm.es (J.M.G.); mguijarro@ucm.es (M.G.); 2 Department of Computer Architecture and Automatic Control, Faculty of Informatics, Complutense University, Madrid 28040, Spain; E-Mail: mmontalvo@fdi.ucm.es; 3 Centre for Automation and Robotics (UPM-CSIC), Arganda del Rey 28500, Madrid, Spain; E-Mails: luis.emmi@car.upm-csic.es (L.E.); pablo.gonzalez@car.upm-csic.es (P.G.-S.)

**Keywords:** weeds detection accuracy, crop lines detection accuracy, camera-based sensor, extrinsic and intrinsic parameters, uncontrolled illumination, lens vignetting correction

## Abstract

In Precision Agriculture, images coming from camera-based sensors are commonly used for weed identification and crop line detection, either to apply specific treatments or for vehicle guidance purposes. Accuracy of identification and detection is an important issue to be addressed in image processing. There are two main types of parameters affecting the accuracy of the images, namely: (a) extrinsic, related to the sensor's positioning in the tractor; (b) intrinsic, related to the sensor specifications, such as CCD resolution, focal length or iris aperture, among others. Moreover, in agricultural applications, the uncontrolled illumination, existing in outdoor environments, is also an important factor affecting the image accuracy. This paper is exclusively focused on two main issues, always with the goal to achieve the highest image accuracy in Precision Agriculture applications, making the following two main contributions: (a) camera sensor arrangement, to adjust extrinsic parameters and (b) design of strategies for controlling the adverse illumination effects.

## 1. Introduction

The use of robotic systems, equipped with vision-based sensors, for site-specific treatments in Precision Agriculture (PA) is seeing continuous growth. A common practice consists of image processing for weed and crop identification. Both, crop lines and weed identification are used for selective treatments [1–13]; additionally crop line identification is also used for tractor guidance [14–17]. Crop line and weed detection is an important issue related with the application of machine vision methods in agriculture, and consequently has attracted numerous studies in the area.

Ratios of greenness to soil determine what is known as density. The goal is always to detect crop/weed densities and also crop lines from the imaged spectral components. These tasks must always be carried out using a given camera-based sensor with the highest accuracy and robustness possible and under adverse and highly variable outdoor illumination conditions, which are the natural environmental conditions in agricultural fields.

Accuracy may be critical for determining whether specific treatments are required for weed control or for vehicle guidance when required. Accuracy in this matter is directly related with intrinsic and extrinsic camera parameters [18], and also with some factors caused by the uncontrolled outdoor illumination [19].

The camera-based sensor consists of three main parts, namely: (a) CCD device; (b) optical lens and (c) ultraviolet and infrared cut filters for controlling the input of only those wavelengths of interest. Some camera parameters are fixed for the system's requirements according to the goals of the application. Sometimes this leads to the choice of a specific sensor, as described below in Section 2, with their intrinsic parameters already predefined and perhaps some extrinsic ones too. This paper is concerned with the definition and analysis of those unfixed extrinsic parameters and also with factors to control the adverse effect of the illumination, always with accuracy purposes for greenness identification and later crop line and weed detection.

Regarding the *extrinsic parameters*, they depend directly on the physical vision system, which is installed on-board the tractor pointing to a selected area in the field, ahead of the tractor. This is an important issue in PA, which is addressed in this paper and described in Section 2.2.1.

Regarding the *illumination factors*, we can point out that the processing of agronomic images becomes a difficult task because they are always captured under uncontrolled illumination conditions [19]. Indeed, in outdoor environments a variety of weather conditions may appear, *i.e*., highly sunny or cloudy days with different intensities, clear days alternating with different cloud densities, *etc*.

Moreover, it is well known that in outdoor environments, particularly in sunny days, infrared radiation enters the sensor impacting the different spectral channels. The control of illumination factors is addressed in Section 2.2.2.

Based on a given camera-based sensor with its corresponding accessories, we study the image accuracy for crop line and weed detection in agronomical images with specific reference to maize crops, where an area 3 m wide must be covered. This accuracy is studied from two points of view, making the main findings of this paper: (a) geometrical arrangement, based on extrinsic parameters and (b) software corrections for improving the image quality, derived from the uncontrolled illumination in this kind of outdoor environments. This paper is organized as follows: Section 2 describes materials and methods used for accuracy determination considering the above two points of view. In Section 3 accuracy results are provided. Finally, Section 4 presents the relevant conclusions.

## 2. Materials and Methods

### 2.1. Materials

**Figure 1.**
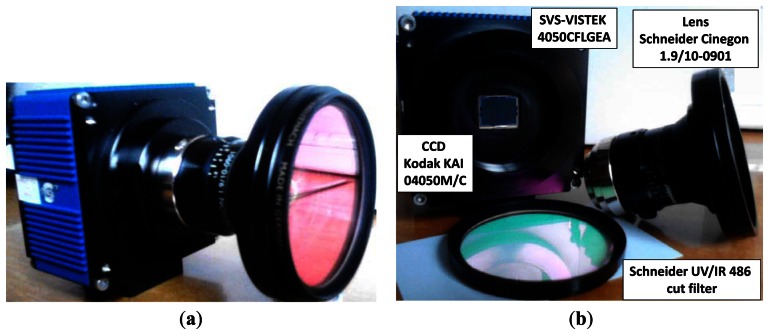
CCD sensor, lens and UV/IR cut filter: (**a**) integrated. (**b**) separated.

The camera-based sensor consists of three essential physical parts: (a) CCD-based device embedded in a housing with its electronic equipment and interfaces for power supply and to the computer; (b) optical lens and (c) ultraviolet and infrared cut filter. Figure 1 displays these parts assembled as a whole in Figure 1(a) and separated in Figure 1(b).

**Figure 2.**
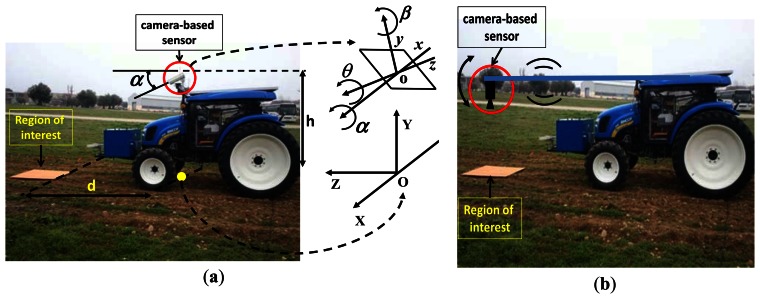
Camera-based sensor arrangement with a ROI in front of the tractor: (**a**) near the mass center of the tractor with referential coordinate systems; (**b**) Zenithal position.

The CCD is a Kodak KAI 04050M/C sensor with a Bayer color filter with GR pattern; resolution of 2,336 × 1,752 pixels and 5.5 × 5.5 μm pixel-size. This device is part of the SVS4050CFLGEA model [20] which is robust enough and very suitable for agricultural applications. This device offers several externally controlled possibilities: (*a*) exposure time, which determines the time taken to capture the image; (*b*) Red, Green and Blue gains, where a value can be set for each channel, including gains auto-calculation; (*c*) definition of specific Regions Of Interest (ROIs); (*d*) information about the operating temperature. This Gigabit Ethernet device connected to a cRIO-9082 with dual-core controller, 1.33 GHz and LX150 FPGA running under LabView 2011 from National Instruments [21] is robust enough and specifically designed for real-time processing, so both features are very suitable for our agricultural application. Because the application occurs in harsh environments (containing dust, drops of liquid from sprayers, *etc*.) it is encapsulated in a housing with IP65 protection and internally equipped with an automatic fan which is triggered if the temperature surpasses 50 °C; this housing is displayed in Figure 2(a) indicated by the label camera-based sensor.

The optical system consists of a Schneider Cinegon 1.9/10-0901 lens [22], with manual iris aperture (f-stop) ranging from 1.9 to 16 and manual lockable focus, providing high stability in the agricultural environment. It is valid for sensor formats up to a diagonal value of 1″, *i.e*., maximum image circle of 16 mm, and is equipped with an F-mount which can be adapted to C-mount. The focal length is fixed at 10 mm. Its field of view is above 50° with object image distance from infinity to 7.5 mm, which allows the mapping of a width of 3 m as required for our application. Its spectral range varies from 400 to 1,000 nm, *i.e*., visible and near-infrared (NIR). Under this optical system the images are captured with perspective projection [18].

As mentioned before, our system works in adverse outdoor agricultural environments where the natural illumination contains a high infra-red component. The sensor is highly sensitive to NIR radiation and to a lesser extent to ultra-violet (UV) radiation. The NIR heavily contaminates the three spectral channels (Red, Green and Blue) producing images with hot colors. This makes identification of crop lines and weeds unfeasible because during the treatments these structures are basically green. To avoid this undesired effect, the system is equipped with a Schneider UV/IR 486 cut-off filter [23]. Its operating curve specifies that wavelengths below 370 nm and above 760 nm are blocked, *i.e*., both UV and NIR radiation. Despite this blocking effect, a vignetting effect remains, requiring correction as described below.

More than 2,000 images have been acquired in the CSIC-CAR facilities in Arganda del Rey (Madrid) on different dates, during April/May/June 2011 from maize fields and the last ones on November 2012 and January 2013. No maize crops are available at this time of the year. Because our application is specifically designed for maize crops, crop lines have been made by mowing six 80 meter long lines among weeds. Lines are separated 75 cm from each other like in real maize crops.

To quantify the number of pixels with the maximum accuracy as possible, a bright orange colored cardboard of 1 × 1 m^2^ is used. This cardboard defines the physical ROI to be imaged with a peculiar color, which is not present in agronomic images. It is placed in front of the tractor at different distances. As mentioned before, these distances define one of the extrinsic parameters involved in this study related to accuracy from a geometric point of view.

### 2.2. Methods

#### 2.2.1. Accuracy from the Point of View of Extrinsic Parameters

As mentioned before, the vision system is installed on-board a tractor pointing to a selected area ahead. Figure 2(a) displays the camera configuration in the tractor, specifically devoted to treatments in maize fields under the project funded under the Seventh Framework Program: RHEA—Robot Fleets for Highly Effective Agriculture and Forestry Management [24]. In this project an important issue is related to the area to be treated, because the implement used for applying treatment, covers four crop lines spaced 0.75 m each, so the total wide area to be captured for the camera must be at least 3 m in width. Although it is always possible to combine different camera resolutions with different focal lengths, an acceptable solution for positioning the camera, at the same time the width of the scene is captured fulfilling this requirement, is the one displayed in Figure 2(a).

Under this configuration, the origin of the world coordinate system *OXYZ* is located exactly in the ground with its axes oriented as displayed. At a height *h* from *O* is placed the origin *o* of the coordinate system *oxyz* attached to the plane of the image, *i.e*., coinciding physically with the CCD. Angles pitch (*α*), roll (*θ*) and yaw (*β*) define the three degrees of freedom of the image plane with respect to the *OXYZ* system. They define three extrinsic parameters, which are the basis for building the rotation matrix. A fourth extrinsic parameter is *h*, which defines the height of the camera; it is involved in the translation matrix. Although the position of the camera is fixed under this tractor configuration, some displacements along the vertical are still possible, so that *h* can be considered variable.

The tractor moves along the crop lines and it processes in real time the images determining the percentages of weeds and the crop line location. Tractor speed is a critical factor since the faster the tractor moves along the crop lines, the faster the algorithms for detection have to be or else the further away the selected area to be focused has to be, with the resulting unavoidable loss of accuracy.

Ideally the best pitch angle would be 90°, *i.e*., with camera pointing downwards (zenith orientation). This camera arrangement has been used for discrimination between crops and weeds in sugar fields [25] and its performance has been reported in [26]. In our application, this pitch angle arrangement would imply that the camera would have to be placed under the tractor. This solution requires a lens which allows a broad field of view to cover the required width of 3 m and that images processed on real time would have to be processed nearly instantaneously. Another solution would be placing the camera fixed on a bar (Figure 1(b)) at a certain distance ahead of the tractor so that algorithms would have more time to process the images as the tractor reaches the processed area. But this performance would produce high oscillations with the tractor's movement which would result in useless blurry images. Therefore, an acceptable position for the camera on the tractor is the one as close to its mass center as possible to avoid oscillations and with the pitch angle adjusted so that the camera points to the ROI with the highest possible accuracy.

In the same way, the shorter the distance of the region of interest to the tractor, *i.e*., to the origin of the *OXYZ*, the higher the accuracy obtained in the acquired images. However, the area must not be too close because of the following two constraints: (*a*) the box in front containing energy accumulators requires a minimum pitch angle to cover the ROI; (*b*) in the time for image processing; the minimum distance is limited since a very short distance to the tractor would not give time enough for image processing.

Regarding the extrinsic roll (*θ*) and yaw (*β*) parameters, we assume they are fixed to zero values because of the plane terrain (roll) and correct guidance (yaw). Obviously, they could be considered to be non-zero if the above constraints are not met. Thus, assuming both extrinsic parameters are known, the image accuracy, based on the above mentioned constraints, depends on the pitch angle (*α*), the height of the camera (*h*) and the distance of the ROI (*d*), so this paper studies the image accuracy based on these three parameters, *i.e*., *α*, *h* and *d*, which are related to each other. The goal is to find out the relation among them to determine the camera arrangement needed to achieve the best image accuracy. This represents an extension with regard the study in [27], where only the pitch angle was the subject of study, also for image accuracy, under a simulated scenario through Webots [28], instead of a real one as now. The use of simulated scenarios is a common practice before using the real ones [9]. Depending on the agronomic application some systems set beforehand some extrinsic parameters because the system performance is guaranteed, either for detecting weeds and crops [29–31] or for row following [32,33], including stereovision systems [34].

**Figure 3.**
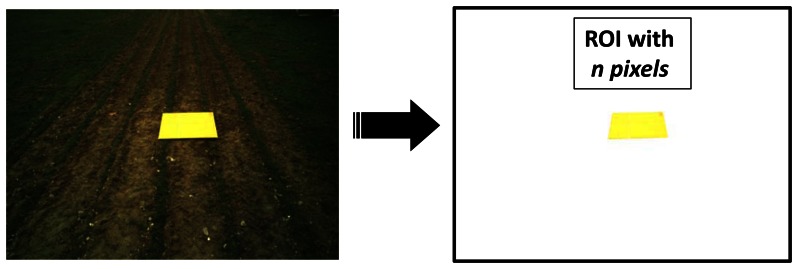
ROI in the field defined by the orange cardboard and number of its pixels.

For quantifying the ROI resolution of every image captured under different values of the extrinsic parameters to be studied, this image is segmented and the number of pixels of the orange cardboard is computed based on a segmentation process developed under LabView. This process is easy, it is based on the color spectral properties displayed by the peculiar orange color mapped on the image from the original cardboard, every non bright orange pixel is removed, so that the remaining ones in the image belong to the ROI, Figure 3. The higher the number of remaining orange pixels in the image the higher the resolution of the ROI. This is the criterion for measuring the accuracy based on the extrinsic parameters.

A set of 125 images have been acquired consisting of five different pitch angles, five different heights and five different distances, all combined with each other. This means that every image corresponds to a different pitch angle, a different height of the sensor camera from the ground and a different distance of the ROI from the sensor camera.

In order to achieve this set of images we proceed as follows:

1. The camera-based sensor is placed in the same position as the one it has in the tractor pointing at the crop lines.
2. The cardboard is placed in the middle of the crop lines at the distance where weeds will be detected once the tractor is in movement (Figure 2).
3. A set of different images is taken varying the pitch angle, the height of the camera from the ground and the distance of the camera to the ROI alternatively. That is:
*for h* = *h*_1_ to *h*_5_ do *for d* = *d*_1_ to *d*_5_ do  *for α* = *α*_1_ to *α*_5_ do
capture image *I_hdα_*identify the cardboard; apply color image segmentation based on the RGB spectral componentscompute the area in terms of the number of pixels belonging to the identified cardboard*end; end; end;*

#### 2.2.2. Accuracy from the Point of View of Illumination Factors

As already mentioned, the quality of the images is highly dependent on illumination, which affects highly the segmentation algorithms and indirectly the image accuracy from the point of view of crop line detection and weed identification. Thus, in addition to the extrinsic parameters, the image accuracy can be studied from the point of view of image quality. In this paper, we make the following contributions to this topic:

- Controlling the amount of light impacting the sensor. The diaphragm or iris aperture is fixed, because no auto-iris lens is available in our system, and the amount of light is controlled via the *exposure time*, which is a facility provided by the camera sensor.
- Applying a software correction procedure for minimizing the phenomenon known as *lens vignetting effect*, where the brightness decreases at the periphery in the image compared to the center of the image. This effect can be produced by different causes, including optical properties of the lens itself, particularly in lenses with wide aperture. When we apply a cutting infrared filter, part of the red wavelengths are attenuated causing an excess of greenness, particularly where vignetting is more evident, *i.e*., at the periphery of the image and more specifically on the four corners.

##### (a) Exposure Time

**Figure 4.**
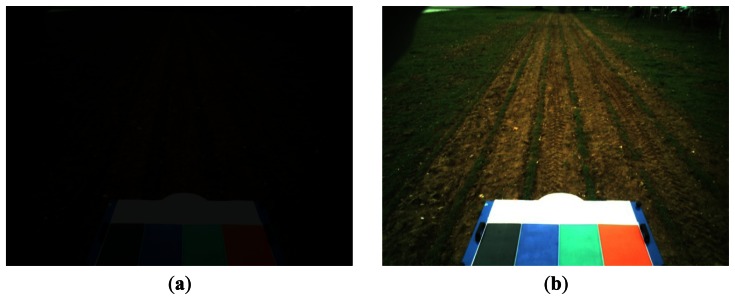
Images captured from the same scene with two different exposure times: (**a**) *E_t_* = 2 × 10^4^ μs; (**b**) *E_t_* = 3.5 × 10^4^ μs.

A panel with four colors is placed in front of the tractor and inside the field of view of the camera. At this stage we assume known the extrinsic parameters for the best accuracy, *i.e*., geometric camera arrangement with *α* and *h* values. Thus, we know the position of the panel in the image and also the distribution of colors on it (Figure 4). The white part of this panel serves as reference for controlling the exposure time.

The procedure consists of the following steps:

1. Capture an image.
2. Perform a sampling inside the white part and compute the average value for the three spectral RGB components, *i.e*., *R̅, G̅*, and *B̅*.
3. Adjust the *Exposure time* (*E_t_*) so that the highest of the three maximum average values, *H* = *max*{*R̅, G̅*, *B̅*}, falls on a given interval to ensure that the sensor is sufficiently excited but without reaching saturation. In our sensor with 8 bits in resolution per channel, the maximum value is *M* = 2^8^ = 255, hence the interval is as follows: *aM* ≤ *H* < *bM*, where *a* and *b* define the lower and upper limits, in our experiments we have verified that *a* = 0.90 and *b* = 0.98 suffices.
4. *E_t_*, which represents the previous exposure time, is adjusted as follows: if *H* < 0.90*M* then *E_t_* = (1+*p*) *E_t_*; if *H* > 0.98 *M E_t_* = (1−*p*)*E_t_* otherwise *E_t_* does not need updating; where 0 < *p* < 1 represents the fraction of adjusting, set to 0.20 in our experiments.
5. If after the adjustment *H* does not fall inside the interval specified, a new image is captured with the last *E_t_* updated and steps *a*) to *(d*) are repeated again.

Figure 4(a) displays an image captured with an *E_t_* = 2 × 10^4^ μs where the white panel displays values requiring *E_t_* adjustment; Figure 4(b) shows the same image acquired with *E_t_* = 3.5 × 10^4^ μs. We can see how the whole image, and particularly the white panel, have increased their values resulting in a brighter image.

##### (b) Vignetting Correction

As specified by the manufacturer, the Schneider UV/IR 486 cut-off filter [23] is based on what is known as thin-film technology containing more than thirty coats on one of its sides and a multi-resistant coating on the opposite one. The incidence angle of these rays in the periphery of the filter is greater than in the center and they must travel longer distances along the different layers of interference. This effect is more pronounced the shorter the focal length of the lens, *i.e*., lenses with wide-angles. This occurs in our case with a lens of 10 mm.

The effect is that the real color is displaced toward green and blue at the expense of the red one. Thus, in order to correct this effect we have designed an image gray pattern (*P*) with values ranging in [0,1], where at the center of the pattern (*c_x_*, *c_y_*) the value is zero and achieves its maximum value of one at the four corners. The size of this pattern is exactly the one of the image and the value of each pixel *i* located at (*x*,*y*) is computed as follows:

(1) $$d(x,y)={\left({\left(x-c_{x}\right)}^{2}+{\left(y-c_{y}\right)}^{2}\right)}^{\frac{1}{2}}$$

**Figure 5.**
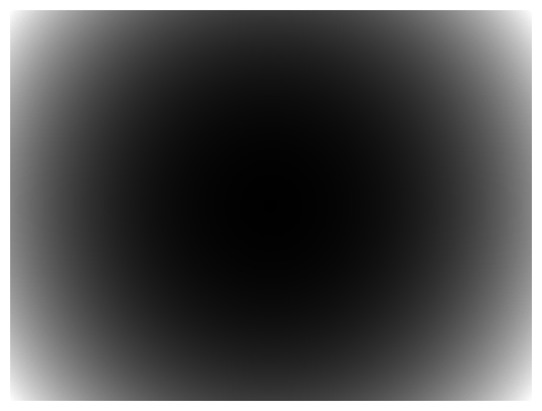
Image pattern used for vignetting correction.

Figure 5 displays this pattern. Given a channel *R*, *G* and *B* we apply the following operation to obtain the corrected values, *R_c_*, *G_c_* and *B_c_*:

(2) $$\begin{array}{ccc} Rc=(1+K_{r}P)\times R; & Gc=(1+K_{g}P)\times G; & Bc=(1+K_{b}P)\times B \end{array}$$

where × denotes pixel-by-pixel multiplication instead matrix product; *K_r_*, *K_g_* and *K_b_* represent the trade-off between corrections, based on the behavior of the Schneider UV/IR 486 cut-off filter [23] we have verified, with high level of satisfaction that the following values are appropriate in our experiments: *K_r_* = 0.3; *K_g_* = 0.0 and *K_b_* = 0.0. This means, that only the red channel is corrected in our application where crop line and weed detection with high accuracy is the goal.

## 3. Results and Discussion

This study is concerned with the accuracy of weed and crop line detection in maize fields under the RHEA project [24]. It has been focused on the definition of three critical extrinsic parameters and also on how to control adverse effects produced by the illumination.

### 3.1. Analysis of Extrinsic Parameters

Table 1 shows the number of pixels (*n*) obtained for the ROI after applying the method described in Section 2.2.1 for the set of 125 images. Each image *I_hdα_* is captured under different values for the pitch angle (*α*), height of the camera (*h*) and distance to the ROI (*d*).

**Figure 6.**
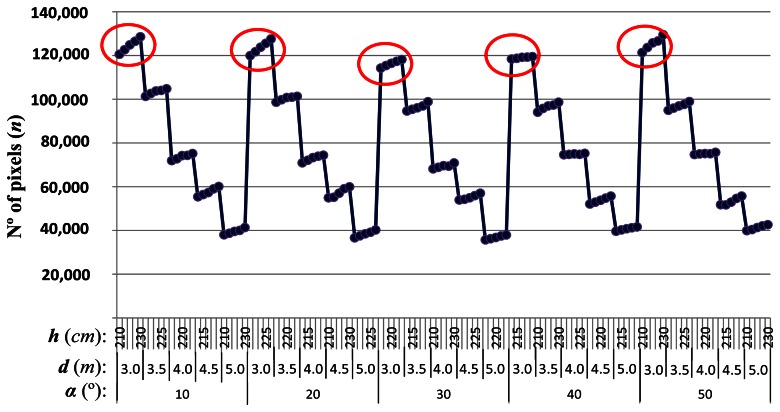
Dependency of the number of pixels (*n*) against *h*, *d* and *α*.

Results in Table 1 are graphically displayed in Figure 6 for convenience and better visibility, with the 125 values obtained for *n*. As we can see from the graph, there are five sets of five values each (marked with a red circle) that correspond to the maximum values of *n*. All these values were obtained at the distance of 3 meters which leads us to the first conclusion of our study (a quite intuitive but perhaps not obvious one): “*The closer the ROI is from our camera the higher the resolution and therefore the accuracy on weeds and crop lines detection*”.

In order to assess the real results in Table 1 against theoretical results, we have developed a simulation program in Matlab [35]. With such purpose in mind, we define a ROI with identical size *i.e*., 1 × 1 m^2^. The coordinate systems are defined like in the real situation and the focal length is also fixed to 10 mm. The CCD-device specifications are the ones corresponding to the Kodak KAI Sensor provided in Section 2.1. The free parameters provided to the program are obviously *h*, *d* and *α*. For each combination of these free parameters we map the four corners from the ROI into the corresponding pixels in the image, obtaining a trapeze. We compute the area of this trapeze and compute the percentage against the number of pixels (*n*) displayed in Table 1. The average value for all combinations of the free parameters in Table 1 is 0.0088% with standard deviation of 7.9 × 10^−4^. As we can see, simulated and real values are very close, verifying the validity of the real experiments for the proposed sensor arrangement and the configuration of the extrinsic parameters.

**Figure 7.**
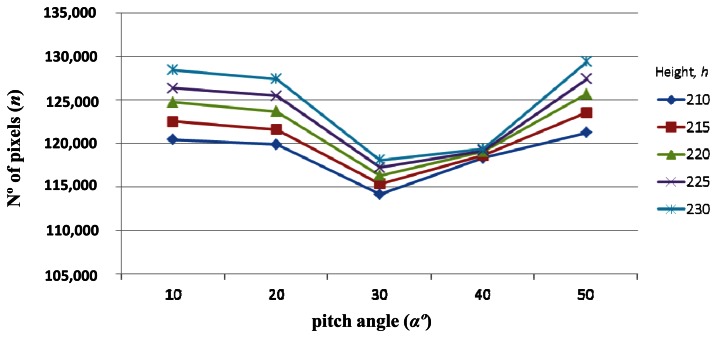
Number of pixels *n*, against *α*, for the five different heights *h*, at a fixed distance *d* = 3 m.

Once we know that distance is a critical parameter for accuracy, we fix the distance of the ROI at 3 m and see how *n* varies with the other two parameters. Fixing distances away from the tractor we graph the different values of *n* depending on the pitch angle (*α*) and the height (*h*) of the camera (Figure 7).

**Figure 8.**
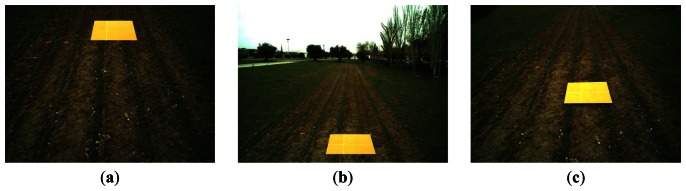
Different images of the ROI (bright orange cardboard) captured with *h* = 215 cm and *d* = 3 m under three different pitch angles: (**a**) *α* = 50°; (**b**) *α* = 10° and (**c**) *α* = 30°.

From Figure 7 we can see that the best pitch angles, in terms of accuracy, are those in which the ROI is placed either at the top of the image which corresponds with *α* = 50° (Figure 8(a)), or at the bottom of the image with *α* = 10° (Figure 8(b)), and the worst pitch angle is that in which the ROI is placed at the center of the image with *α* = 30 (Figure 8(c)). The images in Figure 8 were captured at a height *h* = 215 cm and a distance *d* = 3 m. The above conclusion was already reported in [15], although only considering the pitch angle (*α*) and without the intervention of the height (*h*). Thus under this more exhaustive study we arrive at the same conclusion with respect the pitch angle.

**Figure 9.**
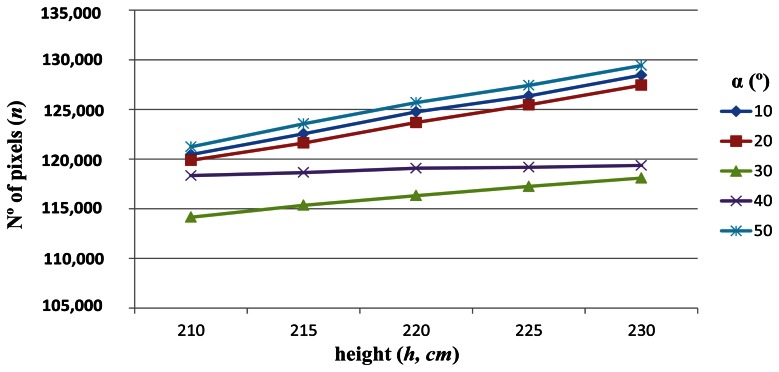
Number of pixels *n*, against *h*, for five different pitch angles *α*, at a fixed distance *d* = 3 m. (dot means Decimal point or thousand, please change to comma if dot refer to thousand, the same to Figure 13).

Regarding the height (*h*) in Figure 7, we can see that the greatest number of pixels is always obtained with *h* = 230 cm. This appears a surprising result because without exhaustive analysis one might feel that the lower height objects appear with greater resolution. To clarify this, we again display the number of pixels, *n* against the pitch angle *α*, and the height *h*, but this time we place on the *x*-axis the height (Figure 9).

**Figure 10.**
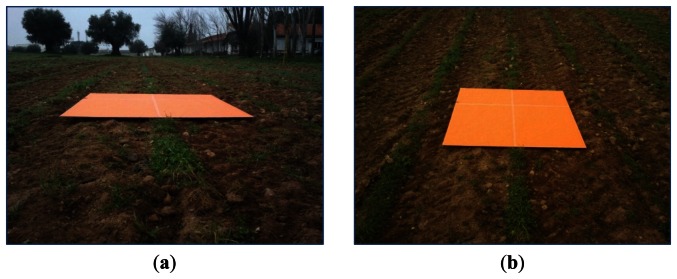
Images of the same ROI at a fixed distance and the same pitch angle: (**a**) with the camera almost at ground level; (**b**) image obtained at a greater height from the ground.

Indeed, we can see that the accuracy increases as we increase both the height of the camera in the tractor and the pitch angle. This is due to the geometric camera arrangement. Figure 10 displays a pedagogical example where this is clarified. If we would place the camera at a very close height from the ground, at a fixed distance *d* with a given pitch angle *α*, we would get an image like the one displayed in Figure 10(a), however as *h* and *α* increase with *d* remaining fixed, due to a new perspective arrangement, the area of the cardboard that can be seen from the camera also increases, Figure 10(b).

To emphasize the importance of choosing the correct parameters in order to get the highest possible accuracy for a specific area of the image, we take a look of the maximum number of pixels, *n_max_* and the minimum, *n_min_*, obtained in our study:

$$\begin{array}{lll} n_{\text{max}} & = & 129,423\;\text{pixels},\left(\alpha =50{}^{\circ},\;h=230\;\text{cm},\;d=3\;\text{m}\right).\\ n_{\text{min}} & = & 35,648\;\text{pixels},\;\left(\alpha =30{}^{\circ},\;h=210\;\text{cm},d=\;5\;\text{m}\right). \end{array}$$

This means that the same area of a region in the 3D scene can be imaged into a ROI with a very different number of pixels depending on the parameters involved in this study, *i.e*., *α*, *h* and *d*. This can be critical in some agricultural applications. For instance, consider a combination of parameters giving *n* = 129,423 and a second arrangement with *n* = 35,648, this results in a loss of resolution up to 72.5%, *i.e*., the accuracy reduction.

We have studied the best arrangement for the extrinsic parameters and we have found a trade-off between them. Considering that the tractor is in movement at a speed of approximately 4 m/s and our ROI is 5 m deep by 4.5 m wide a good choice of parameters is: *α* = 20°, *h* = 220 cm and *d* = 4 m. The parameters we have chosen give us, according to our study, a resolution of *n* = 56,985. If we compare this resolution with the maximum resolution obtained in our study (*n* = 129,423) we realize that, due to constrains not related with the sensor camera, we are giving up 56% of the accuracy. However, had we not considered the importance of these parameters, a worse election of these would have led us to a reduction of 72.5% of the resolution.

So, apart from the intrinsic parameters there are other parameters to be considered with a critical influence on the accuracy of the ROI. This means that when the sensor specification is provided we need to determine, depending on the application, which is the best arrangement of the extrinsic parameters. In this study we have only considered the pitch angle, but also accuracy depends on the yaw and roll angles. These angles should be considered if the camera arrangement involves its consideration or when the tractor must work in a sloping ground.

### 3.2. Analysis of Illumination Factors

**Figure 11.**
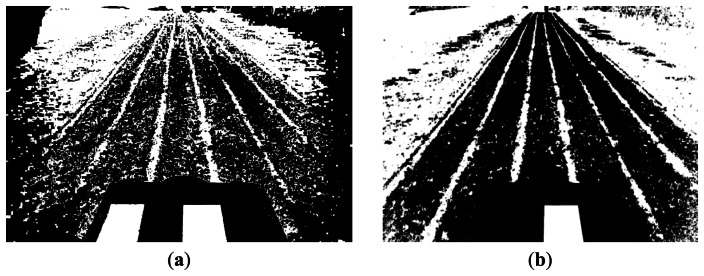
Segmented images obtained from the ones in Figure 4 respectively.

The second group of factors affecting the image accuracy, now in terms of the quality of the image based on the RGB spectral components, is the one derived from the illumination factors. In order to verify the relevance of these factors we apply the segmentation process described in [2], which is based on the computation of a global threshold through a fuzzy clustering learning-based strategy. The images under analysis contain two main classes of interest, *i.e*., vegetation and no vegetation. The fuzzy clustering is designed to compute the cluster centers associated to each of these two classes based on specific samples of the image. These samples are represented by features with the three RGB spectral components. The cluster centers are three dimensional vectors with three spectral values. Once the cluster centers are estimated, a threshold value is obtained as the percentage of the green spectral component with respect the three ones. This threshold allows us to identify green plants from the remaining parts in the images and the corresponding binary image is finally obtained. Figure 11 displays the results of applying this process to the images displayed in Figures 4, respectively, which were obtained with two different exposure times.

**Figure 12.**
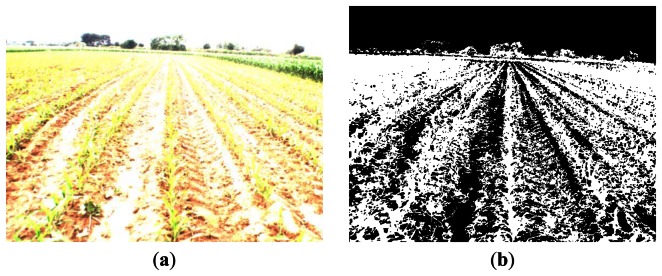
(**a**) Original saturated image; (**b**) binary image after segmentation.

The exposure time for the image in Figure 4(a) was below which it would be desirable, unlike the one obtained in the image in Figure 4(b), which was sufficient. As we can see, the result in the first case is worse than in the second one. Indeed, the binary image displayed in Figure 11(b) contains an over-segmentation in the part of interest, where crop lines and weeds are placed with important gaps on the outer part. A possible explanation to this phenomenon is that the sensor requires sufficient time to be impacted by the reflectance and the illumination coming from objects in the scene. Because there are different types of materials, the reflectance and illumination sent to the sensors is different for each type of material. When the exposure time is insufficient, the sensor produces this kind of effect. On the contrary, if the exposure time is excessive the intensity image becomes saturated and the image segmentation process fails. Figure 12(a) displays a saturated image and its corresponding segmented image in Figure 12(b) using the same segmentation procedure as before; we can see how the result becomes unfeasible. From the point of view of weeds and crop lines detection, this leads to clear inaccuracies.

From the point of view of accuracy, color or white balancing is not required because these processes are only suitable for a correct human perception [36]. This represents an important advantage over other systems that perform this kind of operations.

**Figure 13.**
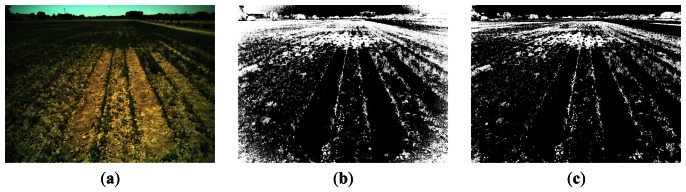
(**a**) Original image; (**b**) binary image without vignetting correction; (**c**) binary image with vignetting correction.

Regarding the process related to vignetting, Figure 13(a) displays an original image and Figure 13(b,c) the binary segmented images without and with vignetting correction. In Figure 13(b) we can easily see how an excess of white pixels appears at the four corners representing green plants, which is not present in the image of Figure 13(c) after vignetting correction. Thus, when no vignetting correction is applied, high inaccuracy results in the corners during weed and crop line detection.

In order to test the performance of the proposed method to control the adverse illumination factors, we have applied the following procedure:

(a) With the tractor stopped at different positions in the field, we acquire an image that needs exposure time adjustment according to the procedure described in Section 2.2.2. A second image is acquired after the adjustment. Thus, for each position two images are acquired and stored as RGB in the BMP format, *i.e*., without compression to avoid losses.
6. Each image of the stored pair is processed with and without vignetting correction. Thus, for each pair we obtain four binary images through the method described in [2], as already mentioned.
7. For each pair of images a ground-truth image is built as follows. From the four binary images obtained above, we select the one with the highest quality according to an expert human criterion by comparing it with the pair of original images. The selected binary image is manually touched up, so that isolated or groups of pixels are relabeled as white or black pixels, also according to the human criterion.
8. The four binary images are compared against the corresponding ground-truth by computing the Correct Classification Percentage (PCC) index [37]:
  (3) $$\text{PCC}=\frac{TW+TB}{TW+TB+FW+FB}$$

where *TW* (true whites) and *TB* (true blacks) are the number of white/black pixels respectively in the image that are also white/black in the ground-truth; *FW* (false whites) and *FB* (false blacks) are the number of white/black pixels respectively in the image that are also black/white in the ground-truth.

We have analyzed 25 pairs of images captured as described in Step (1) above during different days and under different illumination conditions. Table 2 displays the averaged *PCC* values over the 25 binary images obtained after the processing with and without exposure time adjustment and vignetting correction. The analysis is carried out after removing the top third of each image, because this part is out of our specific interest.

As we can see from results in Table 2, the highest percentage is obtained after exposure time adjustment and vignetting correction. This implies that the best accuracies are obtained after the application of these two processes.

## 4. Conclusions

The present study is part of the work developed in the RHEA project [24], where the reason for developing this study was to acquire images with the highest possible accuracy for weed and crop line detection. We have studied two main sources of inaccuracies. The first could be caused because of an incorrect arrangement of the extrinsic parameters, once the intrinsic ones have been set beforehand due to the different requirements derived from the agronomic application at hand. Some of these requirements are that ROI to be treated must be placed at a certain distance so that we have enough time (real-time) to process the image and posterior action for treatment. On the same way, a very high pitch angle may make some of the crop lines of interest disappear from the image. Another question to be considered in our case is that the ROI cannot be placed at the very top or at the very bottom of the image because once the tractor is in movement, irregularities on the ground produce oscillations of the tractor and the camera that may make the ROI disappear from the image. Additionally, depending on each project, it is obvious that in other projects extrinsic and intrinsic parameters can be combined to achieve a trade-off among them for maximum accuracy. The second source is derived from the uncontrolled illumination that causes insufficient or excessive CCD sensor activation, producing infra- and over-saturation. Also the illumination causes the known vignetting effect, when it crosses an UV-IR coated cut-off filter.

We have also proposed solutions for correcting the adverse illumination effects with the goal of maximum accuracy, concluding that for outdoors image processing, illumination must be constantly calibrated in real time as images are taken. There is no “unique” value for exposure time or iris aperture valid for any atmospheric weather conditions.

## Figures and Tables

**Table 1.** Number of pixels (*n*) in the ROI obtained for different combinations in the pitch angle (*α*), height of the camera (*h*) and di*s*tance to the ROI (*d*).

**α = 10°**			**α = 20°**			**α = 30°**			**α = 40°**			**α = 50°**		
*d* (m)	*h* (cm)	*n*	*d* (m)	*h* (cm)	*n*	*d* (m)	*h* (cm)	*n*	*d* (m)	*h* (cm)	*n*	*d* (m)	*h* (cm)	*n*
3.0	210	120,456	3.0	210	119,872	3.0	210	114,152	3.0	210	118,338	3.0	210	121,236
	215	122,547		215	121,622		215	115,351		215	118,645		215	123,568
	220	124,754		220	123,682		220	116,321		220	119,078		220	125,698
	225	126,354		225	125,462		225	117,244		225	119,176		225	127,425
	230	128,452		230	127,442		230	118,089		230	119,357		230	129,423
3.5	210	101,239	3.5	210	98,589	3.5	210	94,523	3.5	210	94,021	3.5	210	94,865
	215	102,569		215	99,745		215	95,325		215	95,685		215	95,862
	220	103,698		220	100,695		220	95,986		220	96,852		220	96,899
	225	103,967		225	100,885		225	96,854		225	97,246		225	97,585
	230	104,693		230	101,210		230	98,752		230	98,563		230	98,865
4.0	210	71,895	4.0	210	70,912	4.0	210	68,108	4.0	210	74,594	4.0	210	74,625
	215	72,698		215	71,992		215	68,812		215	74,729		215	74,987
	220	74,147		220	73,245		220	69,543		220	74,947		220	75,125
	225	74.236		225	73.856		225	69.332		225	74.752		225	75.012
	230	75,100		230	74,342		230	70,811		230	75,219		230	75,652
4.5	210	55,326	4.5	210	54,856	4.5	210	53,865	4.5	210	52,023	4.5	210	51,745
	215	56,314		215	55,123		215	54,169		215	52,869		215	51,658
	220	57,259		220	56,985		220	54,896		220	53,695		220	52,896
	225	58,965		225	59,001		225	55,896		225	54,754		225	54,585
	230	59,996		230	59,896		230	56,987		230	55,625		230	55,632
5.0	210	37,989	5.0	210	36,552	5.0	210	35,648	5.0	210	39,602	5.0	210	39,874
	215	38,654		215	37,472		215	36,182		215	40,226		215	40,325
	220	39,541		220	38,442		220	36,762		220	40,713		220	41,256
	225	39,987		225	39,152		225	37,421		225	41,160		225	42,015
	230	41,253		230	40,232		230	37,895		230	41,581		230	42,569

**Table 2.** Averaged *PCC* values for the images analyzed with and without exposure time adjustment and with and without vignetting correction.

	**Images Requiring Exposure Time Adjustment**		**Images after Exposure Time Adjustment**	
vignetting correction	No	Yes	No	Yes
PCC	66	74	83	91
